# Inguinal Ring RNA Sequencing Reveals Downregulation of Muscular Genes Related to Scrotal Hernia in Pigs

**DOI:** 10.3390/genes11020117

**Published:** 2020-01-21

**Authors:** Gabrieli de Souza Romano, Adriana Mercia Guaratini Ibelli, William Raphael Lorenzetti, Tomás Weber, Jane de Oliveira Peixoto, Mauricio Egídio Cantão, Marcos Antônio Zanella Mores, Nelson Morés, Victor Breno Pedrosa, Luiz Lehmann Coutinho, Mônica Corrêa Ledur

**Affiliations:** 1 Programa de Pós-Graduação em Zootecnia, Universidade Federal da Bahia, Av. Adhemar de Barros, 500-Ondina, Salvador 40170-110, Bahia, Brazil; gabisouza_romano@yahoo.com.br; 2 Embrapa Suínos e Aves, Concórdia, Rodovia BR-153, Km 110, Distrito de Tamanduá, 321, Santa Catarina 89715-899, Brazil; adriana.ibelli@embrapa.br (A.M.G.I.); jane.peixoto@embrapa.br (J.d.O.P.); mauricio.cantao@embrapa.br (M.E.C.); marcos.mores@embrapa.br (M.A.Z.M.); nelson.mores@embrapa.br (N.M.); 3 Programa de Pós-Graduação em Ciências Veterinárias, Universidade Estadual do Centro-Oeste, Alameda Élio Antonio Dalla Vecchia, 838-Vila Carli, Guarapuava 85040-167, Paraná, Brazil; 4 Programa de Pós-Graduação em Zootecnia, UDESC-Oeste, Rua Beloni Trombeta Zanin 680E-Bairro Santo Antônio, Chapecó 89815-630, SC, Brazil; williamlorenzetti20@hotmail.com; 5 BRF SA, Curitiba, PR. Present address: Instituto Federal de Educação, Ciência e Tecnologia do Rio Grande do Sul, Rodovia RS-135, KM 25-Distrito Eng. Luiz, Sertão 99170-000, RS, Brazil; samotweber@hotmail.com; 6 Departamento de Zootecnia, Setor de Ciências Agrárias e Tecnologia, Universidade Estadual de Ponta Grossa, Av. General Carlos Cavalcanti, 4748-Uvaranas, Ponta Grossa 84030-900, Paraná, Brazil; vbpedrosa@uepg.br; 7 Departamento de Zootecnia, Escola Superior de Agricultura Luiz de Queiroz (ESALQ), Universidade de São Paulo, ESALQ/USP, Av. Pádua Dias, 11, Piracicaba 13418-900, São Paulo, Brazil; llcoutinho@usp.br

**Keywords:** transcriptome, congenital disease, differentially expressed genes

## Abstract

Scrotal hernias (SH) are common congenital defects in commercial pigs, characterized by the presence of abdominal contents in the scrotal sac, leading to considerable production and animal welfare losses. Since the etiology of SH remains obscure, we aimed to identify the biological and genetic mechanisms involved in its occurrence through the whole transcriptome analysis of SH affected and unaffected pigs’ inguinal rings. From the 22,452 genes annotated in the pig reference genome, 13,498 were expressed in the inguinal canal tissue. Of those, 703 genes were differentially expressed (DE, FDR < 0.05) between the two groups analyzed being, respectively, 209 genes upregulated and 494 downregulated in the SH-affected group. Thirty-seven significantly overrepresented GO terms related to SH were enriched, and the most relevant biological processes were muscular system, cell differentiation, sarcome reorganization, and myofibril assembly. The calcium signaling, hypertrophic cardiomyopathy, dilated cardiomyopathy, and cardiac muscle contraction were the major pathways possibly involved in the occurrence of the scrotal hernias. The expression profile of the DE genes was associated with the reduction of smooth muscle differentiation, followed by low calcium content in the cell, which could lead to a decreased apoptosis ratio and diminished muscle contraction of the inguinal canal region. We have demonstrated that genes involved with musculature are closely linked to the physiological imbalance predisposing to scrotal hernia. According to our study, the genes *MYBPC1, BOK, SLC25A4, SLC8A3, DES, TPM2, MAP1CL3C*, and *FGF1* were considered strong candidates for future evaluation.

## 1. Introduction

The average economic losses estimated globally in livestock due to animal health are over 20% [1], and the congenital anomalies are significant causes of economic losses. Scrotal hernias are common congenital defects in pigs with frequencies ranging from 0.39% to 9.1% according to the animal breeds and lines [2,3]. This condition occurs when abdominal content is present in the scrotal sac, which affects the production, health and animal welfare, leading to economic losses [4,5].

Anatomical factors, such as failure of involution of the inner inguinal ring, imbalance of collagen proportions, non-obliteration of the *processus vaginalis*, and low muscle contraction, can lead to scrotal hernia [6,7,8,9,10]. Despite the environmental influence, significant additive genetic variances were found for SH [3]. In addition, some studies have identified genomic regions and candidate genes associated with this defect [6,11,12,13]. Although these studies provided important information, the approaches used may have been insufficient to explain the etiology of this defect, since some phenotypes can be caused by differences in gene expression, and no variation in genomic sequences occurs [14].

In this perspective, we aimed to clarify the etiology of scrotal hernia by studying its transcriptional events. Gene expression studies allow a better understanding of metabolic and physiological knowledge of the hernia pathology through functional analysis. Thus, it is possible to associate several genes that influence scrotal hernia to pathways and genetic networks that act together in the biological regulation of this defect [15]. Therefore, this study aimed to identify genes and molecular mechanisms involved in the occurrence of scrotal hernia in pigs through the whole inguinal ring transcriptome analysis of normal and scrotal hernia-affected pigs.

## 2. Material and Methods

### 2.1. Animals and Sample Collection

A total of eight non-castrated Landrace male pigs, with 60 days of age, belonging to the same nucleus farm from a private company located in Santa Catarina State, Brazil, was used in this study. Animals were raised according to the technical recommendations for this line, receiving water and feed ad libitum. The pigs were characterized as normal and affected with scrotal hernia and then transported to the Embrapa Swine and Poultry National Research Center, located in Concórdia, SC, Brazil, for necropsy and sample collection. The euthanasia was carried out by electrocution for 10 s followed by immediate bleeding, according to the practices recommended by the Ethics Committee on Animal Utilization of the Embrapa Swine and Poultry National Research Center (protocol number 011/2014), which follows the international guidelines on animal welfare. Approximately 1 cm of the inguinal ring tissue was collected in circular form from four healthy and four scrotal hernia-affected pigs, immediately stored in liquid nitrogen, and frozen at −80 °C) for subsequent gene expression analysis. The normal pigs were selected from families with no history of scrotal hernia at least in the last three generations.

### 2.2. Total RNA Extraction, Library Preparation and Sequencing

Approximately 100 mg of the inguinal ring tissue was ground using liquid nitrogen and a mortar, mixed to 1 mL Trizol reagent (Invitrogen Life Technologies, Carlsbad, CA, USA) and incubated for 5 min in a room temperature (RT) and 0.2 mL chloroform were added. The tubes were shaken vigorously and centrifuged at 12,000× *g* for 15 min at 4 °C. Approximately 600 μL of the upper phase containing the RNA were transferred for a new microtube, where an equal volume of 70% ethanol was added. At this stage, the total RNA isolation was performed using the Qiagen RNeasy^®^ Mini kit (Qiagen, Hilden, NRW, Germany), following the manufacturer’s instructions.

The total RNA samples were quantified using a Biodrop spectrophotometer (Biodrop, Cambridge, UK) and samples with an OD260:OD280 ratio greater than 1.9 were considered pure. The integrity was confirmed in 1.5% agarose gel and also using an Agilent 2100 bioanalyzer (Agilent Technologies; Santa Clara, CA, USA). Samples with RIN > 8.0 were used for the RNA library preparation with a TruSeq Stranded mRNA Library Prep Kit (Illumina, Inc., San Diego CA, USA) using approximately 2 μg of total RNA starting material, according to the manufacturer’s recommendations. The size of the libraries was evaluated in a Agilent 2100 bioanalyzer (Agilent Technologies, Santa Clara, CA, USA) and quantified by qPCR. Sequencing was performed in an Illumina HiSeq 2500 (lllumina, San Diego CA, USA), producing paired-end sequences (2 × 100 bp) at the Functional Genomics Center, ESALQ, University of São Paulo, Piracicaba, São Paulo, Brazil.

### 2.3. RNA-Sequencing Analyses

For data quality control, low quality sequences (QPhred < 24), short sequences (<70 bp), poly A/T tails, contaminants (phiX), and sequence adapters were removed using SeqyClean software (1.8.11, Ilya Zhbannikov, Durham, NC, USA) [16]. Then, sequences were mapped against the pig reference genome (SusScrofa 11.1, Ensembl 92) using the STAR [17] program (2.7, Alexander Dobin, Oyster Bay, NY, USA). The EdgeR [18] package was used to identify differentially expressed genes between the normal and scrotal hernia-affected pigs. Negative and positive log_2_ fold change (LogFC) indicate, respectively, downregulation and upregulation of genes in the affected compared to the normal group. The *p*-value was adjusted for multiple tests using the false discovery rate (FDR), according to Benjamini-Hochberg. Genes were declared as differentially expressed (DE) when FDR < 0.05 and logFC > 0.5.

### 2.4. Functional Analysis

The list of DE genes between the analyzed groups were submitted to a gene ontology analysis using the DAVID Bioinformatics Resources 6.8 database (accessed on 12 May 2019) [19] to obtain genes functionally related to biological processes and metabolic pathways (KEGG). The REVIGO resource was employed to summarize and visualize the enriched GO terms [20]. The analysis of the interaction among all the DE annotated genes was performed using the STRING database [21]. To determine the function of the transcripts that were specified as new genes, the blast2go software (5.2, BioBam Bioinformatics S.L,Valencia, Spain ) was used [22].

### 2.5. qPCR Confirmation

The validation of the RNA-Seq results was performed using quantitative PCR (qPCR) of the same samples used in the RNA-Seq analysis. The complementary DNA (cDNA) synthesis was performed with the SuperScript III ™ First-Strand Synthesis Supermix Kit (Invitrogen, Carlsbad, CA, USA) using 3 μg of total RNA. Primers were designed in exon-exon junctions with the Primer-Blast online tool [23], using sequences obtained in the Genebank [24] and Ensembl [25] databases for eight selected genes: Myosin binding protein C slow type (*MYBPC1*), desmin (*DES),* actin α 1 skeletal muscle (*ACTA1*), actin γ 2 (*ACTG2*), microtubule associated protein 1 light chain 3 γ (*MAP1LC3C*), glucoronidase β (*GUSB*), calponin 1 (*CNN1*), and fibroblast growth factor 1 (*FGF1*) (Table 1). These genes were selected based on the logFC, FDR and gene function. The primers quality was evaluated in the NetPrimer online software [26]. 

The qPCR reactions were performed in QuantStudio 6 (Applied Biosystems, San Francisco, CA, USA) equipment, in a final volume of 15 μL containing 1 × Maxima Mastermix SYBR Green (Fermentas, Waltham, MA, USA), 0.14 μM for each primer and 2 μL of diluted cDNA. The cycle threshold (Ct) means were collected and the relative quantification analysis was performed using the Relative Expression Software Tool 2009 (REST 2009) [27], which uses a pair wise fixed reallocation randomization test. The normalization was carried out using the *PPIA* (peptidyl-prolyl cis-trans omerase A) and *RPL19* (ribosomal protein L19) reference genes, as described by Lorenzetti et al. [28]. Genes were considered DE when *p* ≤ 0.05.

## 3. Results

### 3.1. Sequencing and Mapping

From the whole transcriptome sequencing of the inguinal ring tissue, an average of 28.10 million reads/sample (paired-end 2 × 100 bp) was generated. Approximately 23.8 million reads/sample were maintained after data quality control. A mean of 97.23% of the reads was mapped to the swine reference genome (SusScrofa 11.1, Ensembl 92).

### 3.2. Differential Gene Expression

A total of 13,498 genes (Table S1) were expressed in the inguinal ring transcriptome, corresponding to 60.12% of the total genes in Sus Scrofa genome version 11.1. Before the differential expression analysis, a multi-dimensional scaling (MDS) analysis was performed using all the transcripts to visualize the profile of the studied groups, demonstrating an evident separation of samples collected between the normal and scrotal hernia-affected pigs (Figure S1). 

In the differentially expressed (DE) analysis, it was possible to verify that 703 genes had different levels of expression (FDR < 0.05) between the normal and affected pigs, of which 494 (70.27%) were downregulated and 209 (29.73%) upregulated in the affected group (Table S2). The top ten up- and downregulated genes in pigs affected with scrotal hernia, according to the logFC (Table 2), were related to regulation of intracellular transport, development and differentiation of cells and muscle structure, and phagocytosis. 

In the qPCR analysis, five of the eight evaluated genes were DE (Table 3) between the normal and scrotal hernia-affected groups. Furthermore, all analyzed genes had the same expression pattern of those obtained in the RNA-Seq results.

### 3.3. Functional Annotation and Pathway Analysis

From the 703 DE genes, 684 were coding genes, the other 19 genes had the biotypes IG_C_gene, IG_V_gene, lincRNA, miRNA, miscRNA, pseudogene, and ribozyme. From the DE genes, 81 (11.52%) were classified as uncharacterized genes. When the sequences of the uncharacterized genes were aligned with databases available in the blast2go (5.2, BioBam Bioinformatics S.L,Valencia, Spain), it was possible to verify that 98.9% of them had similarities with genes already annotated (Table 4, Table S3). Those genes were related to calcium ion binding, muscle contraction, and immune system.

**Figure 1:**
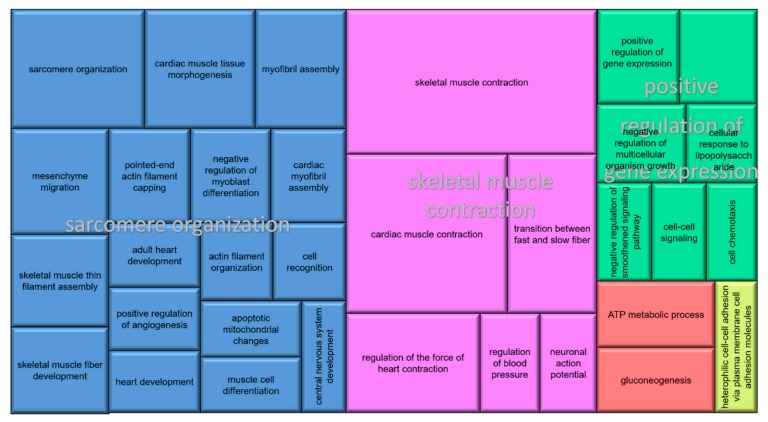
Superclusters of gene ontologies (GO) enriched with differentially expressed genes. Each color indicates a main GO term.

The DE genes were grouped into 37 biological processes (BP), which were associated with five superclusters (Figure 1). From the most enriched GO terms, it was possible to highlight the BPs related to muscular system, including structural muscle development, muscle cell differentiation, sarcome reorganization, myofibril assembly, and ATP metabolic process. Considering the molecular function, it was possible to note the predominance of functions related to signaling, for example, calcium, the cell adhesion molecule, and structural constituent of muscle.

**Figure 2:**
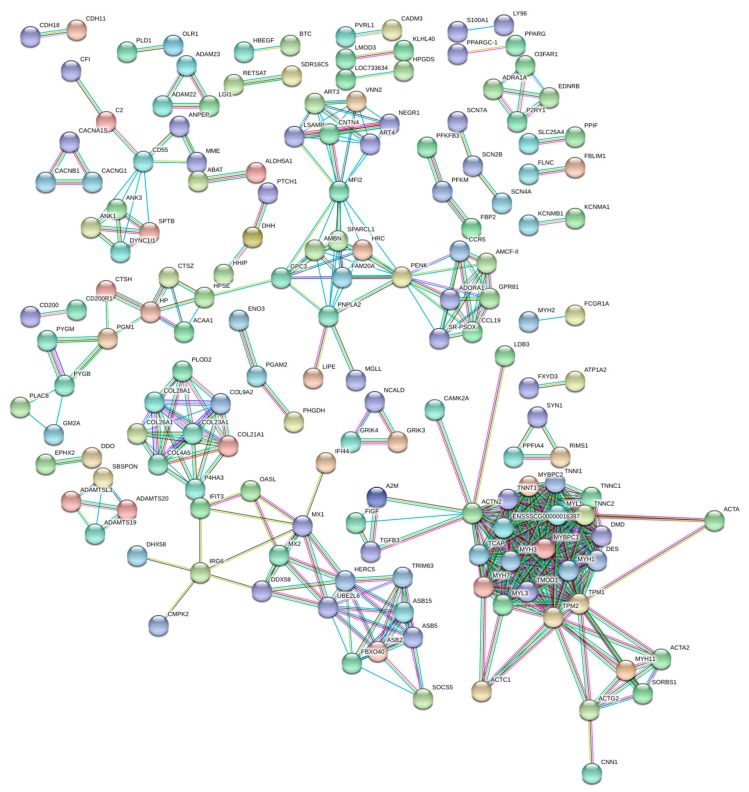
Gene network of DE genes in the inguinal ring tissue from normal and scrotal hernia-affected pigs using STRING. Colored circles represent genes and lines represent the predicted interactions between genes.

The gene network (Figure 2) grouped 31 nodes of major interactions, encompassing genes involved in ion transport, cGMP signaling, muscle contraction, and collagen metabolism. Most of the top DE genes (Table 1) were represented in the interacting gene network. Moreover, there is a link between enriched gene networks and pathways found in the DAVID database, which reinforces the possible influence of these genes in herniation processes (Table 5).

## 4. Discussion

This is the first study that presents a comprehensive transcriptome analysis related to the occurrence of scrotal hernia in pigs using an advanced sequencing strategy. Other studies have pointed out chromosomal regions, candidate genes, and polymorphisms associated with this pathology. However, the genetic mechanisms involved in the etiology of scrotal hernias have not been fully elucidated [11,12,29]. It was possible to observe that the expression profile of several genes in the inguinal ring transcriptome found in this study could explain pathological alterations, such as reduced calcium content in the cell and the presence of smooth muscle, which have already been reported to be involved with scrotal hernia [9,30].

Among the genes with the highest differential expression levels between the affected and normal groups, it is possible to highlight four new genes (Table 4) related to the immune system (*ENSSSCG00000039804*, *ENSSSCG00000036983*, *ENSSSCG00000036318*, and *ENSSSCG00000039102*), characterizing a possible inflammation in the inguinal ring/canal of the animals as a consequence of the hernia development. Clear evidence of inflammatory response in muscle structures of the internal inguinal ring in herniated humans has been previously described [31]. The inflammatory process could be seen as a response to the additional stress of myocytes, due to the steady compressive effect exerted by the abdominal viscera in the inguinal area [32]. In addition, the *GUSB* gene overexpression in animals with scrotal hernia may illustrate the presence of an inflammatory process in the inguinal canal. The *GUSB* gene synthesizes the enzyme β-glucuronidase, essential for the normal restructuring of the extracellular matrix [33]. Increased levels of extracellular β-glucuronidase have been reported in various inflammatory pathologies [34].

### 4.1. Prevalence of Patent Processus Vaginalis

The non-obliteration of the *processus vaginalis* is one of the main factors responsible for the pre-disposition of scrotal hernia and, for this, the apoptosis of the smooth muscle is required [9]. Several downregulated genes in the affected group have important functions in the extrinsic pathways of the cellular apoptosis process, particularly the genes: *AATK* (apoptosis-associated tyrosine kinase), *BOK* (BCL2-related ovarian killer), and *SLC25A4* (solute carrier family 25 member 4) [35,36,37,38,39]. Although these genes have not yet been associated with hernias, the *BAX* gene, from the *BCL2* family, has already been considered a candidate for the occurrence of scrotal hernia in pigs [13]. Moreover, the *MAP1LC3C* (microtubule-associated protein 1 light chain 3), one of the top 10 upregulated genes in the affected group, is involved in autophagous processes [40]. The autophagy process may inhibit the process of apoptosis [41]. Therefore, the deregulation of the previously mentioned genes in scrotal hernia-affected animals may decrease the apoptosis of the smooth muscle, making these animals more susceptible to scrotal hernia.

The obliteration of the *processus vaginalis* and the scrotal hernia are strongly related. The presence of myofibroblasts possibly reflects the attempt of apoptosis by smooth muscle, through cellular differentiation, consisting in an important role in the *processus vaginalis* obliteration [42]. Here, some downregulated genes in the affected group were related to cell differentiation in muscle tissue (Figure 1), such as *MYH11* (myosin, heavy chain 11, smooth muscle), *ACTA2* (actin, α 2, smooth muscle, aorta) and *CNN1* (calponin 1, basic, smooth muscle). Such genes have already been reported as expressed in intermediate stages of differentiation in smooth muscle cells [43,44]. The lower expression of these genes in affected than in normal pigs suggests a reduction in the smooth muscle differentiation and, consequently, failure in the *processus vaginalis* obliteration, resulting in the development of scrotal hernia. 

A significant metabolic pathway found in the transcriptome related to scrotal hernia in pigs was the calcium signaling pathway. Considering only studies with scrotal hernias, there is no previous research reporting genes involved in this signaling pathway. However, decreased calcium levels have been observed in tissues of herniated pigs when compared to normal tissues [30]. The role of ions calcium (Ca^2+^) in the intrinsic pathways of apoptosis signaling has been extensively investigated. There is evidence that the reduction of ions Ca^2+^ in cells limit the activation of apoptosis [45]. In the present study, genes possibly involved in the apoptotic process, such as *SLC8A3* (solute carrier family 8-member A3), *ATP2A1* (ATPase sarcoplasmic/endoplasmic reticulum Ca^2+^ transporting 1), *PLN* (phospholamban), *ADRB1* (adrenoceptor β 1), *CACNA1S* (calcium voltage-gated channel subunit α1 S), and *EDNRB* (endothelin receptor type B) were downregulated in scrotal hernia-affected pigs. The low expression of *ADRB1*, *CACNA1S*, and *EDNRB,* which mediate the entry of ions Ca^2+^ to the intracellular medium, suggests low Ca^2+^ stocks in the cytoplasm, consequently, reflects in the decrease of calcium in the mitochondria [30,46]. It is known that the absorption of Ca^2+^ into the mitochondria is crucial in triggering apoptotic signals [47]. In our study, the downregulation of the *SLC8A3* gene, which encodes a mitochondrial Na^2+^/Ca^2+^ exchange protein [48], was observed. The disturbance of the mitochondrial permeability could prevent the release of cytochrome c into the cytoplasm making apoptosis impossible [9]. This result suggests that the decrease of intracellular and mitochondrial calcium can inhibit the programmed cell death of the smooth muscle cells during the *processus vaginalis* of pigs affected with scrotal hernias. 

### 4.2. Low Muscle Contractile Function in the Inguinal Region

Abrahamson [7] proposed that the presence of a patent *processus vaginalis* does not necessarily indicate the development of an inguinal hernia. Other reasons that can cause the development of scrotal hernia were associated with the obturator and sphincter mechanism, described by Nyhus et al. [49], where the obturator mechanism is needed for the contraction of internal oblique and transverse abdominal muscles for inguinal ring closure, preventing the descent of abdominal contents into the scrotum. On the other hand, when the sphincter mechanism occurs, the contraction is just in the transverse abdominal muscle, resulting in the narrowing of the inguinal ring. In this context, muscle contraction is indispensable, and the committed calcium homeostasis may compromise this metabolic function [50], increasing the susceptibility of pigs to be affected with scrotal hernia.

Additionally, in our study, several muscle genes were DE in the inguinal transcriptome. The gene with the lowest expression in herniated animals, *MYBPC1*, is a crucial component of the sarcomere and important regulator of muscle function [51,52]. Furthermore, interactions of *MYBPC1* with other negatively-regulated genes were observed in pigs affected with scrotal hernia (Figure 2), grouped into metabolic pathways and biological processes responsible for muscle contraction, such as *ACTN2* (α-actinin-2), *DES* (desmin), *MYL1* (myosin light chain 1/3 skeletal muscle isoform), *MYL3* (myosin light chain 3), *TMOD1* (tropomodulin 1), *TNNC1* (troponin C slow skeletal and cardiac muscles), *TNNI1* (troponin I slow skeletal muscle), *TNNT1* (troponin T slow skeletal muscle), *TPM2* (tropomyosin 2 (β)), *ACTA1* (actin, α 1, skeletal muscle), and *ACTG2* (actin, γ 2, smooth muscle, enteric). The proteins encoded by these genes contribute together to the functioning of the muscle contractile machinery [53]. The lower expression of these muscle genes in affected pigs and, consequently, the lower muscle contraction may limit the obturator and sphincter mechanism, predisposing the animals to the development of scrotal hernia.

In this study, some enriched pathways were associated to cardiomyopathy (Table 5), being the first time that these pathways were related to scrotal hernias in pigs. In humans, it has been found that individuals with inguinal hernias also have a predisposition to cardiomyopathies. Therefore, it has been suggested that the etiology of these pathologies could be related, occurring due to the weakness of the connective tissue [54]. Some of the genes that were previously reported to be involved in both pathologies and associated with muscle contraction (*ACTC1, DES, TNNC1, TPM1, TPM2, MYL3*) were DE in our study. Some of those genes have a compensatory response to low contractile function, which may lead to hypertrophic and dilated cardiomyopathy, resulting in myocyte hypertrophy [55,56]. Moreover, the content of embryonic cardiac muscle in the gubernaculum during testicular descent has also been previously reported [57]. Thus, it is suggested that the low expression of the genes associated with cardiac muscle contraction present in the gubernaculum will allow myocyte hypertrophy and, consequently, an increase in the size of this structure, which may enlarge the inguinal ring in a non-physiological way and remain open after the testicular descent.

### 4.3. Changes in Collagen Proportions in the Inguinal Ring

Pathological changes in collagen are also involved in the development of scrotal hernia [58], resulting in weakness of the inguino-abdominal region [59]. In our study, several collagen family genes were DE: six genes were downregulated (*COL23A1*, *COL28A1*, *COL4A5*, *ENSSSCG00000001500*, and *ENSSSCG00000023322*) and two genes were upregulated (*COL8A2* and *COL26A1*) in scrotal hernia-affected pigs. Lower expression of the *COL23A1* gene may cause decreased protein expression of cell adhesion complex proteins, such as integrin [60], making the inguinal ring flexible. Furthermore, *COL23A1* has already been associated to the development of scrotal hernia in pigs [11]. An important paralog of the *ENSSSCG00000023322* gene, the *COL9A1*, has also been described as a potential candidate for scrotal hernia development in pigs [5]. Additionally, the upregulation of the *COL26A1* gene was observed. This gene is involved in the initial development of testis and ovaries as an extracellular matrix component [61]. After birth, the high expression of *COL26A1* may indicate an abnormal dynamic of the remodeling/degradation of the extracellular matrix. These results suggest that the deregulated expression of these collagen family genes in the affected animals may alter the collagen ratio, decreasing the collagen structures in the inguinal tissue, making it more easily degradable. Moreover, it is possible that there would be a decrease in the resistance and increase in the flexibility of the inguinal tissue, favoring the appearance of scrotal hernia.

Although several members of the collagen family were DE, no pathway in the functional analysis described the mechanisms involved in collagen metabolism. Therefore, we investigated a list of DE genes that may be related to the synthesis of this protein and identified genes potentially associated with this condition, such as *HOXC10.* The *HOX* family genes (homeobox) participate in collagen metabolism and are possibly related to the inguinal wall fragility. In this study, *HOXC10* was downregulated in animals affected with scrotal hernia and, although it has not yet been associated with hernias, the *HOXA10* gene, which belongs to the same gene family, has already been considered a candidate for causing this pathology [12].

In addition, it was possible to verify the upregulation of the *FGF1* gene (fibroblast growth factor 1) in herniated animals. There is an evidence that the *FGF1* gene can promote fibroblast proliferation [62]. Tanyel et al. [63] reported the presence of cells morphologically similar to fibroblasts associated with inguinal hernia. Moreover, the *FGF1* gene can inhibit the differentiation of myofibroblasts induced by TGF-β1, regulate the expression of type I collagen, and regulate collagenase-1 [64]. The type I collagen is predominantly found in connective tissues, and is mainly responsible for the tensile strength [10]. Thus, the over-expression of the *FGF1* gene may alter the metabolism of type I collagen, making the inguinal region more flexible promoting the development of scrotal hernia.

The embryonic and fetal development comprises a complex series of well-orchestrated events and when properly performed result in a healthy newborn [65]. According to the results of our study, calcium signaling, hypertrophic cardiomyopathy, dilated cardiomyopathy, and cardiac muscle contraction were the major pathways possibly involved in the occurrence of the scrotal hernia phenotype. Therefore, the possible disproportion of the collagen in the inguinal tissue, the non-obliteration of the *processus vaginalis*, as well as the low contraction of the inguinal region predispose the animals to this anomaly. The functional analyses of the DE genes obtained in this study are in agreement with the events reported in the literature involved with scrotal hernia. It is important to note that the scrotal hernia was already present when the transcriptome was investigated. Therefore, some of the DE genes identified in our study could be more related to the consequence than to the cause of this anomaly. This study pointed out important molecular mechanisms, which contribute to the better understanding of scrotal hernias etiology in pigs. Further investigation is needed to confirm which of the DE genes are probably causing this process.

## 5. Conclusions

In this study, genes related to smooth muscle differentiation, calcium signaling pathways, and apoptosis were DE between normal and scrotal hernia-affected pigs. The expression profile of those genes in affected pigs was associated with the diminished smooth muscle differentiation, followed by low calcium content in the cell, leading to a reduced apoptosis ratio and muscle contraction of the inguinal canal region. We have demonstrated that genes involved with musculature are closely linked to the physiological imbalance predisposing animals to scrotal hernia. According to our study, the *MYBPC1*, *BOK*, *SLC25A4*, *SLC8A3*, *DES*, *TPM2*, *MAP1CL3C*, and *FGF1* genes were considered strong candidates for future evaluation.

## Figures and Tables

**Table 1:** Primers used in the qPCR analysis of the inguinal ring tissue in pigs.

Gene	Primer Sequence	Ensembl ID
*MYBPC1*	F: CAAAAGGGGAGGCTGGAACT	ENSSSCG00000000866
	R: GCCCGACTACTCAAACCTGG	
*DES*	F: ACTTCCGAGAAACAAGCCCT	ENSSSCG00000020785
	R: TGGCTTTAGAGCACCTCGTG	
*ACTA1*	F: TGAAGATCAAGATCATCGCCCC	ENSSSCG00000010190
	R: CAGCTGTTGGAATGGGGTTTAG	
*ACTG2*	F: CCTTCATCGGCATGGAGTCAG	ENSSSCG00000008294
	R: CAGCTGTTGGAATGGGGTTTAG	
*MAP1LC3C*	F: TGGAAACAGCTGGAGGAATGAG	ENSSSCG00000010870
	R: CCTCTCTTCTGGTTGCTAAGCTC	
*GUSB*	F: GACGGACACCTCCAAGTACC	ENSSSCG00000007739
	R: CAGTCCCGCGTAGTTGAAGAA	
*CNN1*	F: TGAGGTCAAGAACAAGCTGGC	ENSSSCG00000013614
	R: GGGTGGACTCATTGACCTTCTTC	
*FGF1*	F: CAGTGACAGCACAGAGCAGA	ENSSSCG00000024954
	R: GGTGCTTTCGAGGCTGAAGA	

F: forward; R: reverse.

**Table 2:** Top 10 downregulated and upregulated genes in pigs affected with scrotal hernia.

Downregulated Genes				
Ensembl Gene ID	Gene Symbol	Description	LogFC	FDR
ENSSSCG00000010190	*ACTA1*	Actin, α skeletal muscle	−13.41	4.80 × 10^−8^
ENSSSCG00000000866	*MYBPC1*	Myosin binding protein C, slow type	−13.28	2.95 × 10^−10^
ENSSSCG00000016157	*MYL1*	Myosin light chain 1/3, skeletal muscle isoform	−12.91	4.82 × 10^−8^
ENSSSCG00000032720	*SMPX*	Small muscular protein	−12.25	4.94 × 10^−7^
ENSSSCG00000014324	*MYOT*	Myotilin	−11.85	2.27 × 10^−7^
ENSSSCG00000031903	*TNNT3*	Troponin T, fast skeletal muscle	−11.61	1.28 × 10^−7^
ENSSSCG00000039710	*MYL2*	Myosin regulatory light chain 2, Ventricular/cardiac muscle isoform	−11.33	1.33 × 10^−8^
ENSSSCG00000013354	*CSRP3*	Cysteine and glycine-rich protein 3	−11.24	6.61 × 10^−7^
ENSSSCG00000011325	*MYL3*	Myosin light chain 3	−10.96	5.96 × 10^−8^
ENSSSCG00000029441	*MYH2*	Myosin-2	−10.78	1.59 × 10^−6^
		**Upregulated Genes**		
**Ensembl Gene ID**	**Gene Symbol**	**Description**	**logFC**	**FDR**
ENSSSCG00000029289		Uncharacterized	7.13	1.51 × 10^−23^
ENSSSCG00000034838	*MAP1LC3C*	Microtubule associated protein 1 light chain 3 γ	6.72	1.96 × 10^−26^
ENSSSCG00000039102		Uncharacterized	5.01	1.04 × 10^−7^
ENSSSCG00000036318		Uncharacterized	4.70	4.15 × 10^−7^
ENSSSCG00000036983		Uncharacterized	4.67	3.20 × 10^−6^
ENSSSCG00000039804		Uncharacterized	4.55	6.71 × 10^−8^
ENSSSCG00000007678	*COL26A1*	Collagen type XXVI α 1 chain	4.54	4.37 × 10^−10^
ENSSSCG00000038719		Uncharacterized	4.53	7.59 × 10^−7^
ENSSSCG00000036203		Uncharacterized	4.44	2.62 × 10^−6^
ENSSSCG00000039111		Uncharacterized	4.42	3.48 × 10^−8^

**Table 3:** Relative expression between normal and scrotal hernia-affected pigs obtained in the RNA-Seq and qPCR studies.

	RNA-Seq		qPCR	
Gene	LogFC	FDR	LogFC	*p*-Value
*MYBPC1*	−13.28	2.95 × 10^−10^	−12.29	0.000
*ACTA1*	−13.41	4.80 × 10^−8^	−13.29	0.105
*ACTG2*	−5.95	9.12 × 10^−8^	−5.97	0.006
*CNN1*	−3.73	4.16 × 10^−8^	−3.68	0.023
*DES*	−7.45	6.09 × 10^−14^	−7.38	0.005
*FGF1*	1.91	3.74 × 10^−3^	1.65	0.067
*GUSB*	1.00	1.58 × 10^−2^	0.71	0.082
*MAP1LC3C*	6.72	1.96 × 10^−26^	8.27	0.023

**Table 4:** Top five downregulated and upregulated uncharacterized genes between normal and scrotal hernia-affected group according to the log_2_FC. The information about alignment, gene description, sequence length (nucleotides), *e*-value, and similarity (sim mean) was obtained in blast2go software (5.2, BioBam Bioinformatics S.L,Valencia, Spain).

Ensembl Gene ID	Description	Length	*e*-Value	Sim Mean	logFC
ENSSSCG00000035429	Hemojuvelin isoform X3	1706	5.00 × 10^−119^	96.77	−9.70
ENSSSCG00000034015	Xin actin-binding repeat-containing protein 1 isoform X1	5511	0	87.7	−9.49
ENSSSCG00000036235	Creatine kinase M-type	252	1.52 × 10^−17^	100	−8.78
ENSSSCG00000015747	Myomesin-2	7217	0	93.38	−7.76
ENSSSCG00000036052	Titin isoform X1	1326	0	98.06	−7.62
ENSSSCG00000039804	Immunoglobulin heavy chain variable region	405	1.29 × 10^−65^	97.2	4.55
ENSSSCG00000036983	IgG heavy chain precursor	987	0	96.07	4.67
ENSSSCG00000036318	IgG heavy chain precursor	327	6.64 × 10^−73^	98.62	4.70
ENSSSCG00000039102	Immunoglobulin kappa Variable region	297	5.24 × 10^−63^	94.09	5.01
ENSSSCG00000029289	Cystatin-9-like	1146	1.71 × 10^−93^	76.56	7.13

**Table 5:** Canonical pathways of differentially expressed genes between normal and scrotal hernia-affected pigs.

Canonical Pathway		Genes	*p*-Value
ssc05410	Hypertrophic cardiomyopathy (HCM)	***TGFB3*** *; TPM1; ACTC1; TPM2; MYL3; TNNC1; PRKAG3; CACNG1; CACNB1; SGCA; DES; CACNA1S*	4.16 × 10^−6^
ssc05414	Dilated cardiomyopathy	***TGFB3*** *; TPM1; ACTC1; TPM2; ADRB1; MYL3; TNNC1; CACNG1; CACNB1; SGCA; DES; CACNA1S*	7.72 × 10^−6^
ssc04261	Adrenergic signaling in cardiomyocytes	*MYH7; TPM1; ACTC1; TPM2; ATP1A2; PLCB4; ADRA1A; ADRB1; MYL3; TNNC1; SCN7A; CACNG1; CACNB1; CACNA1S*	2.64 × 10^−5^
ssc04022	cGMP-PKG signaling pathway	*SLC8A3; ATP1A2; PLCB4; MYLK2; ATP2A1; EDNRB; ADRA1A; PPIF; ADRB1; MYLK; SLC25A4; KCNMB1; MRVI1; ADORA1; CACNA1S*	5.02 × 10^−5^
ssc04260	Cardiac muscle contraction	*MYH7; TPM1; ACTC1; TPM2; ATP1A2; MYL3; TNNC1; CACNG1; CACNB1; CACNA1S*	9.46 × 10^−5^
ssc04152	AMPK signaling pathway	*LIPE; ADRA1A; FBP2; PFKFB3; PPARG; PRKAG3; PFKM; CPT1B; SLC2A4; LEPR; PPARGC1A*	8.12 × 10^−4^
ssc04020	Calcium signaling pathway	*SLC8A3; PLCB4; MYLK2; TNNC2; ATP2A1; EDNRB; ADRA1A; PPIF; ADRB1; TNNC1; MYLK; SLC25A4; CACNA1S*	2.04 × 10^−3^
ssc00250	Alanine, aspartate and glutamate metabolism	*ALDH5A1; GPT2; DDO; ABAT; ASPA*	1.31 × 10^−2^
ssc04923	Regulation of lipolysis in adipocytes	*LIPE; ADRB1; ENSSSCG00000010992; PNPLA2; ADORA1; MGL*	1.51 × 10^−2^
ssc01100	Metabolic pathways	*TST; PIK3C2G; ALDH5A1; ANPEP; **FUT8**; GPT2; AK5; PGM1; **GALNT12**; AK1; PHGDH; AMPD1; PLCB4; **GUSB**; ABAT; **ST3GAL5**; B3GNT2; LPIN1; **CMPK2**; HPGDS; **HPSE**; EPHX2; GAL3ST1; FBP2; ACAA1; **PLD1**; PNPLA2; PC; PYGM; CKMT2; **P4HA3**; CYP27A1; ATP6V0A4; PGAM2; ASPA; ENO3; PFKM; ; CKB; DGKG; PYGB; MGLL; ACSM5*	1.81 × 10^−2^
ssc04910	Insulin signaling pathway	*LIPE; FBP2; PYGM; PRKAG3; PPP1R3A; PPP1R3B; PYGB; SLC2A4; PPARGC1A*	1.93 × 10^−2^
ssc01230	Biosynthesis of amino acids	*GPT2; PHGDH; PC; PGAM2; ENO3; PFKM*	1.98 × 10^−2^
ssc04931	Insulin resistance	*PYGM; PRKAG3; PPP1R3A; CPT1B; PPP1R3B; PYGB; SLC2A4; PPARGC1A*	2.49 × 10^−2^
ssc03320	PPAR signaling pathway	***OLR1*** *; ENSSSCG00000010992; ACAA1; PPARG; CYP27A1; CPT1B*	2.85 × 10^−2^
ssc04270	Vascular smooth muscle contraction	*PLCB4; MYLK2; ADRA1A; ACTA2; MYLK; KCNMB1; MRVI1; CACNA1S*	2.95 × 10^−2^
ssc04922	Glucagon signaling pathway	*PLCB4; PYGM; PRKAG3; PGAM2; CPT1B; PYGB; PPARGC1A*	3.51 × 10^−2^
ssc04921	Oxytocin signaling pathway	*PLCB4; MYLK2; MYLK; CAMK1G; PRKAG3; CACNG1; CACNB1; **KCNJ5**; CACNA1S*	3.91 × 10^−2^
ssc01200	Carbon metabolism	*GPT2; PHGDH; FBP2; PC; PGAM2; ENO3; PFKM*	4.90 × 10^−2^

Upregulated genes in scrotal hernia-affected pigs are shown in bold.

## Data Availability

The datasets used or analyzed during the current study are available from the corresponding author on reasonable request. The transcriptome sequences are available in the SRA database with BioProject number PRJNA350530 and biosample numbers SAMN05941818, SAMN05941817, SAMN05941816, SAMN05941815, SAMN05941814, SAMN05941813, SAMN05941812, and SAMN05941811.

## Supplementary Materials

The following are available online at https://www.mdpi.com/2073-4425/11/2/117/s1, Figure S1: Multi-dimensional scaling (MDS) plot of control (HE06, HE08, HE16, HE18) in blue and scrotal hernia-affected samples (HE09, HE13, HE17, HE19) in red, based on the inguinal tissue transcriptome, Table S1: List of total genes that were identified in the inguinal ring transcriptome of scrotal hernia-affected and healthy pigs, showing ensembl ID, LogFC (log_2_-fold change), logCPM (log_2_ counts-per-million), LR (likelihood ratio statistics), *p*-value, FDR (false discovery rate), name, description and type, chromosome, start and end for each gene and average CPM for normal and affected groups, Table S2: List of expressed values of all the differentially expressed genes between scrotal hernia-affected and healthy pigs, showing ensembl ID, LogFC (log_2_-fold change), logCPM (log_2_ counts-per-million), LR (likelihood ratio statistics), *p*-value, FDR (false discovery rate), name, description and type, Chromosome, and start and end for each downregulated and upregulated gene, Table S3: List of manually annotated novel genes using the blast2go software, followed by its description, sequence length in bp, *e*-value, similarity (sim mean) and log_2_-fold change (logFC).
