# Supplementary figures and images for: Inguinal Ring RNA Sequencing Reveals Downregulation of Muscular Genes Related to Scrotal Hernia in Pigs
Source: Genes (Basel). 2020 Jan 21;11(2):117. doi: 10.3390/genes11020117 (PMC7073996; doi:10.3390/genes11020117)

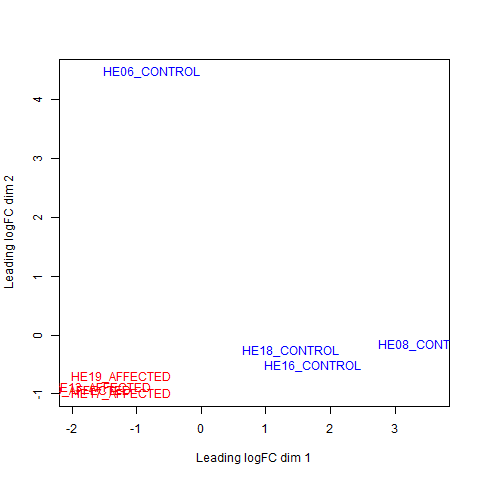

Supplement: Supplementary file 1 [file genes-11-00117-s001.zip › Figure_S1.png]
